# In Vitro Analysis of Heavy Metal Adsorption by Zeolite Skin Care Formulations Using a Quality by Design Approach

**DOI:** 10.3390/ma19040685

**Published:** 2026-02-11

**Authors:** Alessandro Nencioni, Michela Bulfoni, Emanuele Nencioni

**Affiliations:** 1 IBSA Institut Biochimique SA, Via del Piano 29, CH-6915 Pambio Noranco, Switzerland; alessandro.nencioni@ibsa.ch; 2 Department of Medicine, University of Udine, 33100 Udine, UD, Italy; michela.bulfoni@uniud.it; 3 Biofarma Group, Via Castelliere, 2, 33036 Mereto di Tomba, UD, Italy

**Keywords:** zeolite, heavy metal adsorption, skin care formulation, Franz diffusion cell, Quality by Design (QbD)

## Abstract

Zeolites are microporous aluminosilicate minerals widely recognized for their adsorption and ion-exchange properties. Their capacity to capture toxic heavy metals has prompted growing interest in their use as anti-pollution agents in skin care formulations. This study investigates zeolite-based creams through an in vitro permeation test using Franz diffusion cells within a Quality by Design (QbD) framework. A 2 × 2 × 2 full factorial design was applied to evaluate the effects of three critical factors: membrane type (Strat-M^®^ vs. silicone), dosage (10 vs. 20 mg), and dosage regimen. The adsorption and retention of five heavy metals, cadmium (Cd), cobalt (Co), chromium (Cr), lead (Pb), and nickel (Ni), were assessed over 12 h using an in vitro membrane model. The cream containing Zeolite demonstrated significantly higher adsorption of Cr, Co, and Cd compared to placebo and membrane controls, while Ni and Pb exhibited less consistent patterns. No sampling of the receptor compartment was performed; therefore, the analysis focused on metal residues in the donor and membrane compartments. Statistical analyses confirmed the significance of these findings, and graphical trends further supported zeolite’s selective adsorption behavior. Overall, the results provide mechanistic and statistical evidence supporting zeolite as a promising active ingredient for the development of anti-pollution skin care formulations and offer a methodological framework for assessing metal adsorption in topical products.

## 1. Introduction

Environmental pollution represents a growing global concern, with airborne toxicants, particularly heavy metals, posing direct and cumulative risks to human skin [1,2,3]. Chronic exposure to cadmium (Cd), cobalt (Co), chromium (Cr), lead (Pb), and nickel (Ni) has been associated with premature skin aging, oxidative stress, inflammatory processes, and carcinogenic effects [2,4,5,6,7]. Although the stratum corneum constitutes a highly specialized defense system designed to prevent the ingress of exogenous substances, its protective capacity is not absolute [5,8]. Factors such as prolonged environmental exposure, increased concentration of pollutants, compromised skin integrity, and the physicochemical characteristics of individual metals can facilitate penetration into deeper epidermal and even dermal layers [5,6,8,9,10]. Once internalized, these metal ions may bind to cellular proteins, disturb redox homeostasis, alter membrane function, and activate pro-inflammatory signaling pathways [3,6,11]. The resulting cascade of biological events underscores the urgency of exploring new cosmetic and dermatological approaches aimed at reinforcing the skin’s natural defenses and neutralizing hazardous contaminants before they trigger deleterious effects [4,5,6,11]. In response to this need, the cosmetic industry has increasingly integrated the concept of “anti-pollution” into formulation design, seeking active ingredients capable of adsorbing, chelating, or otherwise inactivating toxic metals at the skin interface [4,12,13]. Among the various candidates investigated, zeolites have emerged as particularly promising materials. These naturally occurring or synthetically engineered crystalline aluminosilicates are characterized by a highly ordered microporous architecture, conferring a remarkable capacity for selective ion exchange and molecular adsorption. Their negatively charged framework and large surface area facilitate strong interactions with divalent and trivalent cations, including Cd^2+^, Co^2+^, Ni^2+^, and other environmentally relevant metals, allowing them to effectively trap pollutants through well-defined physicochemical mechanisms [5,12,13,14,15,16,17]. Previous studies have provided preliminary evidence of the suitability of zeolites for skin-protective applications; however, these efforts have often focused on qualitative outcomes, lacking comprehensive experimental design, quantitative rigor, and standardized testing conditions [11,14,17].

To address these gaps, the present study proposes the development of a systematic and reproducible in vitro methodology to quantify the heavy metal adsorption capability of zeolite-based skincare formulations. By combining the structured principles of Quality by Design (QbD) with the Franz diffusion cell system, an established model for evaluating adsorption activity of heavy metals, the research aims to dissect the influence of critical experimental variables such as membrane type, dosing level, and contact regimen [18,19,20]. The QbD framework enables a rational, statistically supported exploration of factor–response relationships, ensuring that the resulting method is not only robust but also sensitive enough to discriminate among formulations with subtle differences in adsorption performance [18,19]. Meanwhile, the Franz cell configuration provides a physiologically relevant environment that simulates real-world exposure scenarios, thus improving the translational value of the findings.

The aim of this study was to provide an in vitro proof-of-concept evaluation of a zeolite-containing cosmetic formulation in a pre-commercial development phase, focusing on the ability of the finished product to retain heavy metals under controlled and reproducible conditions rather than testing zeolite as a raw material alone. Through this integrated strategy, the study provides mechanistic insights into zeolite–metal interactions and contributes to the rational design of effective anti-pollution skin care products aimed at protecting the skin from environmental stressors.

## 2. Materials and Methods

### 2.1. Reagents

The experimental materials included a zeolite-based cream formulation and its corresponding placebo, two synthetic membranes (*Strat-M^®^ and medical grade silicone membrane 15 cm* × *20 cm* × *0.025 cm-* Bioplexus, Boston, MA, USA), and a mixed aqueous metal solution containing cadmium (Cd), cobalt (Co), chromium (Cr), lead (Pb), and nickel (Ni) at a concentration of 0.04 ppm. All reagents and components were obtained from certified commercial suppliers and used as received. PBS components (Na_2_HPO_4_, KH_2_PO_4_, NaCl, H_3_PO_4_) are all from Supelco, Bellefonte, PA, USA, and the Strat-M^®^ membrane is from Merck Millipore (Burlington, MA, USA).

The formulation used in this study was a development prototype and contains herbal-derived ingredients (e.g., plant waxes and Rosmarinus officinalis leaf extract) that may naturally contain trace amounts of heavy metals. Details on the specific composition are reported in Supplementary Table S1.

The zeolite content in the formulation was 3 g per 100 g of cream, and the lipophilic phase represented 17.3% of the total formulation.

The formulation is an emulsion designed to provide a protective barrier effect by forming a continuous film on the skin, which acts as a shield against the adhesion and penetration of environmental pollutants and, in combination with appropriate cleansing procedures, facilitates their removal at the end of the day, thereby promoting deep skin purification.

The overall experimental setup was designed to reproduce physiological skin conditions as closely as possible.

### 2.2. Franz Cell System

#### 2.2.1. Experiment 1: Metals Adsorption Screening

Metals adsorption by zeolite was initially investigated using vertical Franz diffusion cells, each consisting of a donor and a receptor compartment separated by a synthetic membrane. The zeolite-based cream or placebo was applied to the donor compartment, while the receptor compartment was filled with a diluted acidic metal solution designed to mimic the ionic composition of interstitial skin fluid. This setup served as a preliminary screening to assess the adsorption capacity of the zeolite compared to placebo.

#### 2.2.2. Experiment 2: Permeation Study Under Physiological Conditions

In the second experiment, the receptor compartment contained phosphate-buffered saline (PBS) at pH 7.4 (Na_2_HPO_4_ 5.98 g/L, KH_2_PO_4_ 0.19 g/L, NaCl 8.8 g/L, adjusted to pH 7.4 with H_3_PO_4_), while the donor compartment was filled directly with the acidic diluted metal solution in contact with the membrane. This design allowed the evaluation of metals adsorption by zeolite under conditions more closely resembling physiological ionic composition and pH.

In both experiments, temperature of the receptor phase was maintained at 32 ± 1 °C throughout the experiment to simulate the surface temperature of human skin. Continuous magnetic stirring ensured homogeneity and maintained sink conditions over the test duration. Samples from the receptor phase were collected at predetermined time intervals for subsequent analysis of metal content.

### 2.3. Quantification of Metals by ICP-OES

The concentrations of cadmium (Cd), cobalt (Co), chromium (Cr), lead (Pb), and nickel (Ni) in the donor and receptor compartments were quantified using inductively coupled plasma–optical emission spectroscopy (ICP-OES). Samples were first acidified with ultrapure nitric acid (2–5% *v*/*v*) to ensure complete metal solubilization, then filtered (0.22 µm) and transferred into trace-metal-free tubes. Calibration curves for each analyte were generated using certified multi-element standards covering the expected concentration range. ICP-OES measurements were performed under optimized plasma conditions, and emission wavelengths were selected to minimize spectral interferences. Metal concentrations were finally calculated from the calibration models and corrected for dilution factors to obtain the actual levels present in each sample.

### 2.4. Statistical Analysis

Adsorption data were statistically analyzed using one-way analysis of variance (ANOVA), followed by Tukey’s post hoc test for pairwise comparisons. Model adequacy and predictive performance were evaluated through the coefficient of determination (R^2^). The global optimization of experimental conditions was further refined using a desirability function approach to identify the most favorable combination of factors for maximizing metal adsorption by the zeolite-based formulation. Statistical elaboration was performed by MINITAB^®^ 19.2020.1.

## 3. Results

### 3.1. Experimental Design

**Figure 1:**
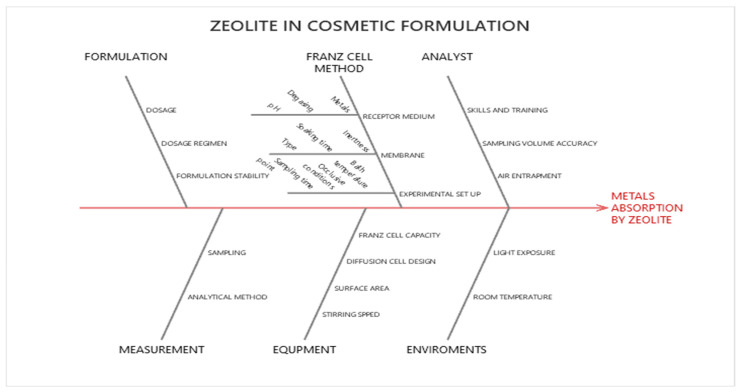
Ishikawa diagram to analytical target development of an in vitro for zeolite absorption.

The overall methodological workflow was structured according to the principles of analytical Quality by Design (aQbD). The process began with the definition of the Analytical Target Profile (ATP), which outlines the performance requirements the method must meet, and with the identification of the Critical Analytical Attributes (CAAs) together with the Critical Method Variables (CMVs), summarized in Table 1 and visually mapped in the Ishikawa diagram (Figure 1). This preliminary phase allowed a clear understanding of which method outputs are essential and which experimental factors are most likely to influence them.

Following this, a formal risk assessment was carried out, applying a Design of Experiment (DoE) approach to systematically evaluate the potential impact of each variable. This step enabled prioritization of factors requiring experimental investigation and guided the development of an efficient study design.

Finally, through response surface analysis of the DoE results, a design space was established, defining the multidimensional combination of method parameters that ensure robust and reliable performance. This structured aQbD pathway provided a rational foundation for method optimization and long-term control [20].

The Ishikawa diagram in Figure 1 provides a structured overview of all potential factors that may influence the metal adsorption capability of zeolite within a cosmetic formulation. It groups the sources of variability into key categories—formulation, Franz cell method, analyst, measurement, equipment, and environmental conditions—highlighting how elements such as dosage, membrane type, sampling accuracy, cell design, and temperature can collectively affect the outcome. This schematic representation supports a systematic identification of critical variables and serves as a foundation for the subsequent risk assessment within the QbD framework.

A full 2 × 2 × 2 factorial design was employed to systematically explore how three critical factors influence the efficiency of metal adsorption. The first factor, membrane type, compared Strat-M^®^ and silicone membranes, chosen because they exhibit markedly different permeability properties and therefore represent complementary models of the stratum corneum barrier. The second factor, dosage, involved testing two application levels, 10 mg and 20 mg, to mimic realistic variations in product use and to assess whether the amount of formulation applied affects adsorption behavior. The third factor, dosage regimen, was evaluated under finite dosing conditions to closely replicate typical topical application scenarios.

By structuring the experiment in this way, the design allowed not only the quantification of the main effects of each variable but also the assessment of their potential interactions, in line with the principles of the Quality by Design (QbD) framework. The full factorial structure also supports the exploration of quadratic response surfaces and the development of second-order polynomial models using the MINITAB statistical software. The independent variables and their corresponding levels, along with the dependent responses considered in the study, are summarized in Table 2.

MINITAB was used to construct the experimental design, optimize the levels of the independent variables, and assess the interactions among the selected process parameters. For generation of the central composite design (CCD), the experimental ranges of each factor were defined using an alpha value of 2, resulting in eight factorial points complemented by four axial points, for a total of twelve experimental runs. This configuration ensured adequate curvature detection and allowed reliable modeling of the response surfaces. All experimental runs, along with their corresponding outcomes, are summarized in Table 3.

The outcomes of the twelve experimental runs generated through the CCD and reveal distinct trends in metal adsorption efficiency as a function of membrane type, dosage, and dosage regimen. Overall, silicone membranes exhibited consistently higher residual metal levels in the receptor phase compared to Strat-M^®^, indicating a lower metal adsorption efficiency by the zeolite formulation when diffusion occurred through silicone. In contrast, Strat-M^®^ runs—particularly those combining high dosage (20 mg) with a finite dosage regimen—showed markedly reduced concentrations of Cr, Co, Cd, Ni, and Pb, reflecting enhanced adsorption and retention of metals by the formulation on the donor side.

Among the tested conditions, Run 3 and Run 11 (Strat-M^®^, 20 mg, finite regimen) demonstrated the lowest metal permeation values across all metals measured, with notable decreases in Cr (0.169–0.232), Co (0.214–0.248), and Cd (0.227–0.285). Conversely, silicone-based runs at the higher dosage level frequently presented the highest metal concentrations in the receptor medium, as observed in Runs 7 and 10, suggesting that membrane characteristics strongly modulate metal transport and adsorption dynamics.

The influence of dosage was also evident: higher application levels tended to reduce metal permeation when combined with Strat-M^®^, whereas the same effect was less pronounced or absent with silicone membranes. The finite dosage regimen (value = 3) generally resulted in lower receptor-phase concentrations than the infinite regimen (value = 0), confirming its suitability for simulating realistic application conditions and for enhancing discriminatory power in method development.

Together, these results highlight the significant impact of membrane selection, formulation dosage, and application regimen on the metal adsorption performance of the zeolite-based cosmetic formulation, thereby supporting the relevance of these factors within the QbD framework.

### 3.2. Optimization Variables with Desirability Function

To achieve simultaneous minimization of all dependent responses, the critical method variables (CMVs) were optimized using the desirability function approach. In this framework, each response is transformed into an individual desirability value ranging from 0 to 1, where 0 indicates a completely unacceptable outcome and 1 represents an ideal or target value. The overall desirability (D) is then calculated as the weighted geometric mean of the individual desirability, providing an integrated measure of how well a given set of experimental conditions meets all objectives. A non-zero D value indicates that all responses fall within acceptable limits, while values approaching 1 reflect highly optimal performance across the full response set. The optimization analysis yielded a maximum desirability of 0.89, indicating an excellent global fit between the targeted and observed outcomes. This optimal condition corresponded to the combination of a Strat-M^®^ membrane, a finite dosage regimen, and a formulation dosage of 30 mg, which collectively minimized the permeation of all monitored metals. The desirability analysis provided a comprehensive visualization of how each response variable contributed to the overall optimization of the method. The composite desirability reached a value of 0.8851, indicating that the selected combination of factors closely aligned with the predefined optimization criteria. The individual desirability plots for Pb, Ni, Cd, Co, and Cr demonstrate that all metals achieved values within the acceptable minimum range, with desirability (d) values consistently above 0.82 and reaching up to 0.95 for Pb. These high desirability scores reflect effective minimization of metal permeation under the optimized conditions. The corresponding factor levels, Strat-M^®^ membrane, a finite dosage regimen, and a 20 mg dosage, are highlighted as the settings that jointly maximize method performance. Together, these results confirm that the model successfully identified an operational window in which metal adsorption by the zeolite formulation is maximized, meeting the global optimization target defined in the QbD framework.

### 3.3. Results of Experiment 1: Direct Adsorption in Acidic Conditions

The first experiment aimed to quantify the direct adsorption capacity of the zeolite-based cream when exposed to an acidic heavy-metal solution, reproducing the chemical environment typically associated with sweat–pollutant interactions. Samples of the metal solution containing known initial concentrations of Pb, Cd, Co, Cr, and Ni were mixed with equal amounts of either zeolite cream or placebo cream, and the residual metal content was measured after 24 h of incubation.

Across all tested metals, the zeolite cream demonstrated a markedly higher adsorption capacity compared with the placebo formulation. Residual concentrations in the zeolite-treated samples were consistently and significantly lower, indicating efficient binding of metal ions under acidic conditions. In contrast, the placebo cream showed minimal adsorption, with metal levels remaining close to the initial concentrations of the stock solution.

Statistical comparison between groups confirmed the difference in adsorption performance, with significant reductions observed for all metals in the zeolite-treated samples. These findings establish that the zeolite-based cream is highly effective at sequestering heavy metals in an acidic environment, providing a baseline reference for evaluating its behavior under more physiologically relevant conditions in Experiment 2.

#### 3.3.1. Metal-Specific Response Patterns

A closer examination of the individual metal profiles highlights the distinct behavior of each element within the system. Chromium (Cr) displayed the most pronounced adsorption in the presence of the zeolite formulation, suggesting a strong binding affinity likely driven by electrostatic interactions between Cr^3+^ ions and the negatively charged sites of the aluminosilicate lattice. Cobalt (Co) followed a similar trend, showing a substantial increase in adsorption when zeolite was present, further confirming the material’s suitability for capturing divalent cations. Cadmium (Cd) also exhibited consistent and marked adsorption by the zeolite-based cream, in agreement with literature that describes a high zeolite affinity for soft metal cations. Nickel (Ni) showed only a modest increase in adsorption compared with the placebo, and this difference did not reach statistical significance, possibly due to competitive interactions among metal ions or restricted access to available adsorption sites. Lead (Pb) behaved the least consistently, a result that may be linked to its lower hydration energy and weaker coordination with zeolitic oxygen sites; moreover, its elevated concentrations relative to the initial diluted acidic solution likely reflect the unexpectedly high Pb content already present in the placebo. Altogether, these findings indicate that zeolite exhibits a selective adsorption mechanism, with strong efficiency toward certain transition metals and more limited interaction with others.

#### 3.3.2. Comparative Performance of Formulations

When comparing the overall performance of the different formulations, the zeolite-based cream consistently outperformed both the placebo cream and membrane-only controls in terms of total metal removal. This was particularly apparent for Cr, Co, and Cd, where residual metal concentrations in the receptor phase were markedly lower following application of the zeolite formulation. The presence of zeolite thus appears to create a barrier-like effect at the membrane interface, effectively sequestering metal ions and reducing their diffusion. The mean residual metal concentrations after 12 h for each tested condition demonstrate the superior adsorption capacity of the zeolite formulation, with significantly reduced levels of Cr, Co, and Cd compared to the placebo and control membranes, whereas Pb and Ni remain largely unaffected.

Statistical analyses corroborated the observed trends. One-way ANOVA revealed significant differences between zeolite and placebo formulations for Cr (*p* = 0.026), Co (*p* < 0.001), and Cd (*p* = 0.004), confirming that the adsorption enhancement provided by zeolite is statistically robust. In contrast, Pb (*p* = 0.147) and Ni (*p* = 0.58) did not reach statistical significance, reflecting greater variability and weaker affinity toward these metals. Tukey’s post hoc comparisons further confirmed the consistency of these results across replicates. The calculated coefficients of determination (R^2^) for the model exceeded 0.9 for Cr, Co, and Cd, indicating excellent model fit and reproducibility of adsorption outcomes. Overall, the statistical evaluation supports the conclusion that zeolite’s adsorption capacity is metal-dependent and more effective toward transition metals with higher charge density and smaller ionic radii.

### 3.4. Results of Experiment 2: Membrane Permeation Study Under Physiological Conditions

A second set of experiments was carried out to evaluate metal adsorption by the zeolite-based cream under conditions more closely resembling physiological skin permeation. Twelve Franz diffusion cells equipped with Strat-M^®^ membranes were used, with six cells treated with zeolite cream and six with placebo cream.

Each donor compartment was loaded with 20 mg of cream (zeolite or placebo), after which 0.4 mL of the diluted acidic heavy-metal solution was applied to simulate topical exposure on pre-treated skin. The donor chambers were sealed, and the permeation study was conducted for 12 h. The receptor chamber contained PBS pH 7.4 (Na_2_HPO_4_ 5.98 g/L, KH_2_PO_4_ 0.19 g/L, NaCl 8.8 g/L, adjusted with H_3_PO_4_), although no receptor sampling was performed, as the objective of this experiment was to quantify the residual metal content in the membrane and donor phase rather than metal permeation.

After 12 h, each Strat-M^®^ membrane together with the donor-phase residue (cream + metal solution) was collected and analysed. The concentrations of Pb, Cd, Co, Cr, and Ni measured in each membrane–donor system are reported in Table 4.

Statistical analysis (one-way ANOVA) revealed significant differences between zeolite-treated and placebo-treated groups for chromium (F(11) = 26.88, *p* < 0.001), cobalt (F(11) = 127.4, *p* < 0.001), and nickel (F(11) = 35.59, *p* < 0.001), demonstrating a markedly greater retention of these metals in the presence of zeolite cream. Cadmium showed a tendency toward significance (F(11) = 11.03, *p* = 0.08), while lead did not differ significantly between treatments (F(11) = 4.6, *p* = 0.58).

Overall, these results indicate that, under buffered and skin-mimicking conditions (Strat-M^®^ + PBS), the zeolite-based cream maintains a robust adsorption capacity for specific metals, particularly Cr, Co, and Ni, confirming its ability to sequester metal ions and limit their permeation through a physiologically relevant membrane model.

## 4. Discussion

The findings of this study confirm the superior capacity of zeolite to absorb specific heavy metals when incorporated into topical formulations, reinforcing its role as a functional ingredient in anti-pollution skin care [13,17]. Among the metals tested, chromium (Cr), cobalt (Co), and cadmium (Cd) exhibited the most significant reduction in permeation, while nickel (Ni) and lead (Pb) showed less consistent trends. This selective adsorption behavior is consistent with previous studies reporting zeolite’s higher affinity toward transition metals with smaller hydrated ionic radii and higher charge density [13,14,17]. Zeolite’s negatively charged aluminosilicate framework favors electrostatic interactions and cation-exchange processes involving divalent and trivalent cations, which explains the preferential binding of Cr^3+^, Co^2+^, and Cd^2+^. The limited adsorption observed for Pb and Ni may be attributed to differences in ionic potential and hydration energy, which affect their ability to access the internal pores of the zeolite lattice. Furthermore, at the slightly acidic pH conditions used in this study, lead tends to form weakly soluble complexes that could limit its exchange with the zeolite matrix [17]. Nickel, on the other hand, is known to exhibit strong affinity for organic functional groups present in the cream base, potentially competing with zeolite for available binding sites. These mechanistic insights suggest that zeolite’s performance can be fine-tuned by modifying pH, surface pre-treatment, or formulation matrix composition to enhance selectivity toward target metals.

The Pb levels detected in the placebo may be related to trace amounts naturally present in some herbal-derived raw materials used in this prototype formulation as indicated by the suppliers’ specifications (<10 ppm for Pb and Ni). While these background levels may influence the absolute reference values, the comparative adsorption trend between placebo and cream containing zeolite remained consistent across experiments, supporting the robustness of the observed effects.

Based on the comparison between the placebo and the zeolite-containing formulation, it can be estimated that approximately 23% of the observed adsorption is specifically attributable to the presence of zeolite; although this represents an approximate calculation, it provides a useful indication of the contribution of zeolite to the overall adsorption capacity of the formulation.

The application of the Quality by Design (QbD) framework proved essential in identifying and controlling critical experimental factors [18,19,20]. The factorial design revealed that membrane type and dosage were the most influential variables affecting adsorption efficiency. Strat-M^®^ membranes provided more reproducible and physiologically relevant results compared with silicone membranes, in line with literature indicating that Strat-M^®^ more accurately mimics the lipid and protein composition of human stratum corneum [8,10]. The use of finite dosing at 20 mg further improved experimental reproducibility and better reflected realistic topical application conditions. These outcomes emphasize the value of Analytical Quality by Design (AQbD) principles in cosmetic formulation testing [18,19,20], offering a statistically robust and reproducible framework for method optimization.

The second set of experiments, conducted under more physiologically relevant conditions using Strat-M^®^ membranes and phosphate-buffered saline (PBS, pH 7.4) in the receptor chamber, further confirmed the metal-specific adsorption capacity of zeolite. In twelve Franz diffusion cells, zeolite cream consistently demonstrated greater retention of Cr, Co, and Ni within the donor compartment and membrane compared with placebo cream, whereas Pb and Cd showed less pronounced differences. These results indicate that, even under buffered and skin-mimicking conditions, the zeolite-based cream maintains a robust adsorption capacity for specific metals, particularly Cr, Co, and Ni, confirming its ability to sequester metal ions and limit their potential permeation through a physiologically relevant barrier.

From a mechanistic standpoint, the enhanced adsorption observed for Cr, Co, and Cd can be ascribed to surface cation-exchange, driven by electrostatic interactions between the zeolite surface and hydrated metal ions. The formation of a zeolite-rich layer on the skin surface may act as a transient “metal scavenger”, preventing penetration of pollutants and reducing their bioavailability. These findings collectively support the concept of zeolite-based creams as physical–chemical barriers that complement other anti-pollution strategies in modern cosmetic formulations, providing a scientifically grounded approach for minimizing exposure to harmful environmental metals.

## 5. Conclusions

This study provides strong and quantitative evidence that cream containing zeolite can selectively adsorb toxic heavy metals, particularly chromium, cobalt, and cadmium, thereby mitigating potential skin exposure to environmental pollutants. The integration of the Quality by Design (QbD) framework with the Franz diffusion cell methodology ensured systematic evaluation of critical parameters, membrane type, dosage, and regimen, resulting in a reproducible and statistically validated in vitro model.

The demonstrated metal selectivity highlights zeolite’s promise as a multifunctional active ingredient for anti-pollution cosmetic formulations. These findings contribute to establishing standardized methods for assessing pollutant adsorption in topical products and support the broader application of QbD principles in cosmetic method development.

The novelty of this work lies in the use of a Franz cell-based in vitro model to investigate the functional performance of the complete formulation, taking into account the influence of the formulation matrix, the membrane interface, and application conditions, thereby providing an early and scientifically grounded validation of the product’s protective potential prior to clinical or market deployment.

Future research should focus on (i) long-term stability and compatibility of zeolite in complex cosmetic matrices, (ii) in vivo validation of metal adsorption and skin protection efficacy, and (iii) potential synergistic combinations with other chelating or antioxidant agents to enhance protective performance against multifactorial environmental stressors.

## Figures and Tables

**Table 1:** Analytical Target Profile (ATP) for the optimization of in vitro method with membrane to adsorption metals.

ATP Element	Target	Scientific Rationale
Active element	Zeolite	Absorption metals. Antipollution action of zeolite
Sample	Zeolite semisolid dosage form	Zeolite formulated in cream was applied to the skin or different membrane to demonstrate their action to sequestrate metals. This action was demonstrated by Franz cell system.
Analytical technique	In vitro method with Franz cell system	Development of an in vitro method with Franz cell system and synthetic membrane to assess the metal adsorption by zeolite formulated in cream.
Applications	Metal absorption behavior assessment	Franz cell system was especially developed to study the topical delivery by mimicking in vitro conditions (temperature, relative humidity and sink condition). Each diffusion cell is constituted by a donor compartment that entails the receptor solution and a membrane, from synthetic or biological origin, that separated both chambers. Formulation was placed on membrane.
Critical Analytical Attributes (CAA)	Amount expressed in ppm of metals absorbed by the zeolite present in the semi-solid formulation.	These CAA should reflect the maximization of the absorption behavior of zeolite.

**Table 2:** Experimental domain of factorial design.

Factors	Level Used	
	Level − 1	Level + 1
Membrane	Silicone	Strat-M^®^
Dosage	0	3
Dosage regimen	10	20

**Table 3:** Experimental variables and results. Data of metal concentrations is reported in ppm.

Run	Membrane	Dosage Regimen	Dosage	Cr	Co	Cd	Ni	Pb
1	Strat-M^®^	10	3	0.325	0.385	0.415	0.367	0.385
2	Silicone	20	3	0.398	0.387	0.399	0.34	0.358
3	Strat-M^®^	20	3	0.169	0.214	0.227	0.237	0.289
4	Strat-M^®^	10	0	0.353	0.451	0.441	0.404	0.396
5	Silicone	10	3	0.336	0.428	0.431	0.403	0.404
6	Silicone	10	0	0.338	0.424	0.436	0.371	0.393
7	Silicone	20	0	0.405	0.411	0.397	0.373	0.313
8	Strat-M^®^	20	0	0.396	0.403	0.406	0.395	0.345
9	Silicone	10	3	0.347	0.417	0.425	0.408	0.412
10	Silicone	20	3	0.405	0.385	0.389	0.414	0.397
11	Strat-M^®^	20	3	0.232	0.248	0.285	0.299	0.302
12	Strat-M^®^	10	3	0.329	0.388	0.402	0.389	0.393

**Table 4:** Residual concentrations (ppm) of Pb, Cd, Co, Cr and Ni detected in the donor compartment (cream + membrane) after 12 h in Experiment 2.

	Pb (ppm)		Cd (ppm)		Co (ppm)		Cr (ppm)		Ni (ppm)	
	Zeolite	Placebo	Zeolite	Placebo	Zeolite	Placebo	Zeolite	Placebo	Zeolite	Placebo
Cell 1A plac + memb		2.423		0.561		0.589		0.810		0.515
Cell 2A zeol + memb	1.270		0.35		0.377		0.582		0.405	
Cell 3A plac + memb		1.308		0.816		0.569		0.653		0.546
Cell 4A zeol + memb	1.137		0.465		0.416		0.526		0.454	
Cell 5A plac + memb		1.83		0.517		0.523		0.666		0.548
Cell 6A zeol + memb	1.039		0.477		0.381		0.541		0.473	
Cell 1B zeol + memb	0.906		0.462		0.329		0.498		0.453	
Cell 2B plac + memb		1.078		0.466		0.570		0.626		0.535
Cell 3B zeol + memb	0.908		0.333		0.384		0.534		0.497	
Cell 4B plac + memb		1.242		0.605		0.547		0.777		0.576
Cell 5B zeol + memb	0.896		0.375		0.411		0.532		0.441	
Cell 6B plac + memb		1.374		0.613		0.564		0.812		0.579

## Data Availability

The original contributions presented in this study are included in the article/Supplementary Material. Further inquiries can be directed to the corresponding author.

## Supplementary Materials

The following supporting information can be downloaded at: https://www.mdpi.com/article/10.3390/ma19040685/s1, Table S1: Specific composition of the prototype cream containing zeolite.
