# Supplementary material for: In Vitro Analysis of Heavy Metal Adsorption by Zeolite Skin Care Formulations Using a Quality by Design Approach
Source: Materials (Basel). 2026 Feb 11;19(4):685. doi: 10.3390/ma19040685 (PMC12942069; doi:10.3390/ma19040685)
Supplement: Supplementary file 1 [file materials-19-00685-s001.zip › materials-4067881-supplementary.pdf]

Supplementary Table S1: Specific composition of the prototype cream containing zeolite.

Water	Solvent
Xanthan Gum	Thickening agent, suspending and stabilizing agent
Pentylene Glycol	Skin conditioning—Miscellaneous
Glycerine	Skin conditioning—Miscellaneous; Humectant
Hialuronic Acid	Anti-aging, Moisturizing, Antioxidant
Caprylic Triglyceride	Emollient
Wheat Germ Oil	Skin conditioning agent—Emollient
Shea Butter	Skin conditioning agent—Miscellaneous, Emollient and Moisturizer
Cetearyl Alcohol	Thickening agent
Cetyl Alcohol	Thickening agent
Mixture of Candelilla, Jojoba, Rice Bran, Polyglyceryl-3-Esters, Glyceryl Stearate, Cerearyl Alcohol and Sodium Stearoyl Lactylate.	Anionic emulsifier
Potassium Cetyl Phosphate	Emulsifier
Glyceryl Undecylenate	Antimicrobial, Emollient and Emulsifier
Rosmarinus officinalis Leaf extract	antioxidant
Zeolite Powder	
Bibasic Potassium Phosphate	pH stabilizer
Potassium Phosphate Monobasic	pH stabilizer
